# Supplementary material for: Fully Automated Photoplethysmography-Based Wearable Atrial Fibrillation Screening in a Hospital Setting
Source: Diagnostics (Basel). 2025 May 14;15(10):1233. doi: 10.3390/diagnostics15101233 (PMC12110636; doi:10.3390/diagnostics15101233)
Supplement: Supplementary file 1 [file diagnostics-15-01233-s001.zip › diagnostics-3533889-supplementary.pdf]

Supplementary Material

Table S1: Extended characteristics of all study participants, grouped in participants with and without atrial arrhythmia including diagnosis and medication at baseline

	Total (n = 346)	Without AA (n = 334)	With AA (n = 12)	P-value
**Age (years)**				0.063[1]
Mean ± SD	72.0 ± 9.8	71.8 ± 9.8	76.9 (10.2)	
Median (IQR)	73 (12)	72.5 (12)	79.5 (11.5)	
Range	32 - 96	32 - 96	58 - 89	
**Sex**				0.738[2]
female	175 (50.6 %)	170 (50.9 %)	5 (41.7 %)	
male	171 (49.4 %)	164 (49.1 %)	7 (58.3 %)	
**BMI**				0.708[1]
Mean ± SD	26.7 ± 5.5	26.8 ± 5.5	26.0 ± 5.2	
**CHA_2DS_2-VASc score**				0.207[1]
Mean ± SD	3.5 ± 1.3	3.5 ± 1.3	4.0 ± 1.5	
Median (IQR)	3 (2)	3 (2)	3.5 (2.25)	
Range	2 – 9	2 – 9	2 - 6	
2 (%)	92 (26.59 %)	90 (26.9 %)	2 (16.7 %)	
3 (%)	111 (32.08 %)	107 (32.0 %)	4 (33.3%)	
4 (%)	72 (20.81 %)	71 (21.3 %)	1 (8.3 %)	
5 (%)	40 (11.56 %)	38 (11.4 %)	2 (16.7 %)	
6 (%)	24 (6.94 %)	21 (6.3 %)	3 (25.0 %)	
7 (%)	4 (1.16 %)	4 (1.2 %)	0	

8 (%)	1 (0.29 %)	1 (0.3 %)	0	
9 (%)	2 (0.58 %)	2 (0.6 %)	0	
**Tattoo in sensor area**	1 (0.3 %)	1 (0.3 %)	0	-
**Skin colour Fitzpatrick scale**[4]				0.908[1]
Mean ± SD	2.2 ± 0.7	2.2 ± 0.7	2.2 ± 0.6	
1 (%)	41 (11.85 %)	40 (12.0 %)	1 (8.3 %)	
2 (%)	217 (62.72 %)	209 (62.6 %)	8 (66.7 %)	
3 (%)	80 (23.12 %)	77 (23.1 %)	3 (25 %)	
4 (%)	2 (0.58 %)	2 (0.6 %)	0	
5 (%)	4 (1.16 %)	4 (1.2 %)	0	
6 (%)	2 (0.58 %)	2 (0.6 %)	0	
**Hairiness in sensor area**[5]				0.832[1]
Mean ± SD	1.3 ± 0.5	1.3 ± 0.5	1.3 ± 0.5	
Median (IQR)	1 (1)	1 (1)	1 (1)	
Range	1 - 3	1 - 3	1 - 2	
1 (%)	241 (69.7 %)	233 (69.8 %)	8 (66.7 %)	
2 (%)	103 (29.8 %)	99 (29.6 %)	4 (33.3 %)	
3 (%)	2 (0.6 %)	2 (0.6 %)	0	
**EHRA score**				
1	-	-	10 (83.3 %)	
2	-	-	2 (16.7 %)	
3	-	-	0	
4	-	-	0	

**Congestive heart failure**				
Yes (%)	42 (12.1 %)	37 (11.1 %)	5 (41.7 %)	0.009[3]
**Hypertension**				
Yes (%)	220 (63.6 %)	213 (63.8 %)	7 (58.3 %)	0.764[3]
**Diabetes mellitus**				
Yes (%)	107 (30.9 %)	102 (30.5 %)	5 (41.7 %)	0.525[3]
**Thromboembolism**				
Yes (%)	20 (5.8 %)	19 (5.7 %)	1 (8.3 %)	0.516[3]
**Vascular disease**				
Yes (%)	144 (41.6 %)	139 (41.6 %)	5 (41.7 %)	1.0[3]
**Angiotensin receptor blocker**				
Yes (%)	82 (23.7 %)	78 (23.4 %)	4 (33.3 %)	0.489[3]
**Aldosterone antagonist**				
Yes (%)	13 (3.8 %)	13 (3.9 %)	0	-
**Diuretic**				
Yes (%)	104 (30.1 %)	100 (29.9 %)	4 (33.3 %)	0.758[3]
**Alpha blocker**				
Yes (%)	12 (3.5 %)	12 (3.6 %)	0	-
**Calcium channel blocker**				
Yes (%)	119 (34.4 %)	115 (34.4 %)	4 (33.3 %)	1.0[3]
**Beta blocker**				
Yes (%)	96 (27.7 %)	93 (27.8 %)	3 (25.0 %)	1.0[3]
**Aspirin**				

Yes (%)	154 (44.5 %)	148 (44.3 %)	6 (50.0 %)	0.772[3]
**Clopidogrel**				
Yes (%)	20 (5.7 %)	20 (6.0 %)	0	-
**Prasugrel**				
Yes (%)	0	0	0	-
**Tricagrelor**				
Yes (%)	7 (2.0 %)	7 (2.1 %)	0	-
**Ticlopedine**				
Yes (%)	0	0	0	-
**Dipyridamole**				
Yes (%)	0	0	0	-
**Statin**				
Yes (%)	151 (43.6 %)	146 (43.7 %)	5 (41.7 %)	1.0[3]
**Insulin**				
Yes (%)	52 (15.0 %)	51 (15.3 %)	1 (8.3 %)	1.0[3]
**Hypoglycemic agents**				
Yes (%)	68 (19.7 %)	65 (19.5 %)	3 (25 %)	0.710[3]

[1] Wilcoxon rank sum test, [2] Chi-squared test, [3] Fisher's exact test, [4] One to Six (pale white to dark brown/black), [5] One to three (moderate to intense hairiness)

Table S2: Differences in compliant and incompliant population

	Compliant (n = 304)	Incompliant (n = 42)	P-value
**Age (years)**			0.502[1]
Mean ± SD	72.2 ± 10.0	71.1 ± 8.5	
Median (IQR)	73.0 (12.0)	70.5 (10.75)	

Range	32 - 96	49 - 89	
**Sex**			0.161[2]
female	149 (49.0 %)	26 (61.9 %)	
male	155 (51.0 %)	16 (38.1 %)	
**CHA_2DS_2-VASc score**			0.954[3]
Mean ± SD	3.5 ± 1.3	3.5 ± 1.4	
Median (IQR)	3 (2)	3 (1.75)	
**Body mass index**			0.573[3]
Mean ± SD	26.7 ± 5.5	27.1 ± 5.1	
**AA incidence**			0.168[4]
Yes (%)	9 (3.0 %)	3 (7.1 %)	
No (%)	295 (97.0 %)	39 (92.9 %)	

[1] t-test, [2] Chi-squared test, [3] Wilcoxon rank sum test, [4] Fisher's exact test

No statistically significant differences were found between the two groups.

Figure S1: Odds ratio forest plot

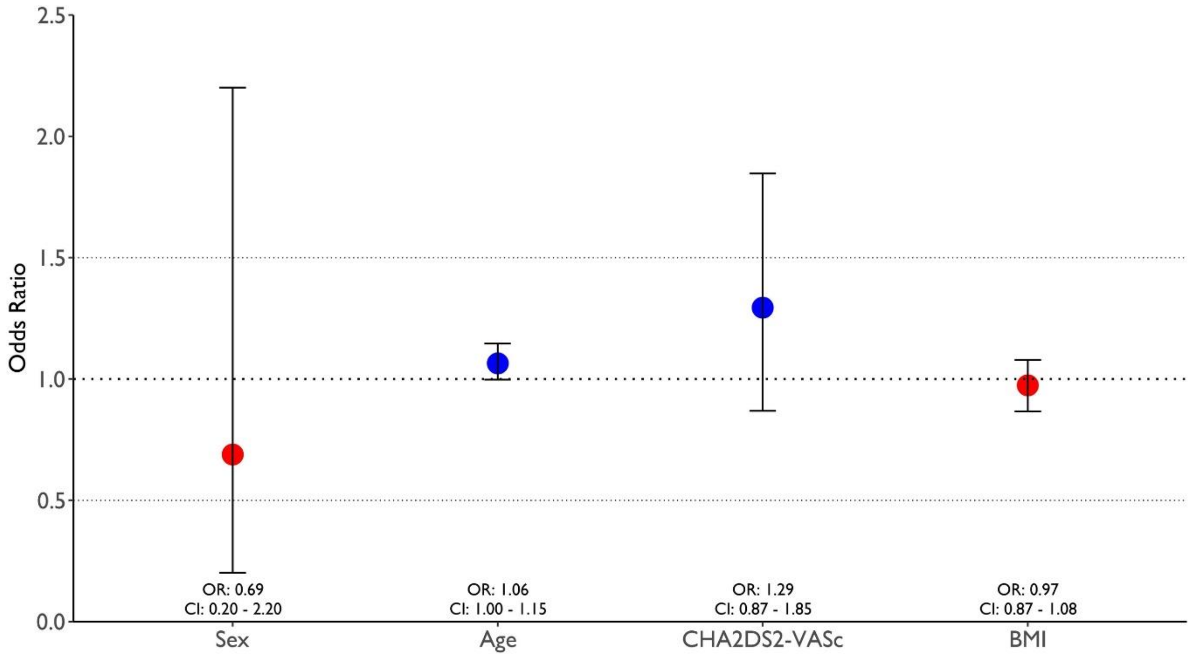

Forest plot of factors associated with AA. Each variable with its corresponding odds ratio (OR) and 95% confidence interval (CI). The red points indicate odds ratio lower than one and blue points higher than one. None of the variables was statistically significant. The variable age had the lowest p-value with 0.076.

BMI, Body Mass Index; Sex, male vs female.
